# Chimeric antigen receptor T cell therapy for the treatment of systemic rheumatic diseases: a comprehensive review of recent literature

**DOI:** 10.1097/MS9.0000000000000891

**Published:** 2023-06-05

**Authors:** Sambhawana Bhandari, Sadikshya Bhandari, Samikshya Bhandari

**Affiliations:** a Danbury Hospital Nuvance Health, Danbury, Connecticut, USA; b Dhulikhel Hospital, Kathmandu University School of Medical Sciences, Dhulikhel, Kavre; c Nepal Medical College Teaching Hospital, Kathmandu, Nepal

**Keywords:** chimeric antigen receptor T cell therapy, rheumatoid arthritis, systemic lupus erythematosus, systemic rheumatic diseases, systemic sclerosis

## Abstract

Systemic rheumatoid diseases (SRDs) are autoimmune and inflammatory disorders that affect multiple organ systems, impacting patients’ quality of life, and survival rates. Standard treatment requires continuous drug therapy and immunosuppression. Chimeric antigen receptor (CAR) T cell therapy has the potential to target and eliminate pathologically activated immune cells and re-establish tolerance in organs affected by dysregulated immunity, making them a promising treatment option for autoimmune diseases. In autoimmune diseases, CAR T cells have the advantage of being able to kill B cells effectively without the need for an accessory cell type. Additionally, CAR T cells targeting CD19 have shown promise in comprehensive B cell aplasia, preserving pre-existing humoral immunity, and specifically eliminating pathogenic B cells. CAR T cell therapy’s limited use in SRDs is due to its inability to effectively target the various autoreactive lymphocytes present. Researchers are developing a universal CAR T cell therapy that detects and targets autoreactive lymphocytes using major epitope peptides, though further studies are required. Moreover, adoptive transfer of CAR-Tregs has shown promise for effectively reducing inflammation and treating autoimmunity. Through this exploration, the authors hope to provide a comprehensive understanding of the current state of research on this topic, identify areas for further study, and promote the advancement of CAR T cell therapy as a treatment option for SRDs.

## Introduction

Highlights

- Immunosuppression is the basis of treatment for the management of rheumatoid diseases.
- Chimeric antigen receptor (CAR) T cells can treat autoimmune diseases by targeting overactive immune cells.
- Universal CAR T cell therapy can target autoreactive lymphocytes with one treatment.
- CAR T therapy targets antigens precisely, minimizing side effects.
- Optimizing the use of CAR T needs study of design, dosing, delivery, and cost.

Systemic rheumatic diseases (SRDs) are chronic inflammatory and autoimmune disorders that affect multiple organ systems in varying degrees of severity and significantly affect patient’s quality of life and overall survival rates^1,2^. These diseases are characterized by the presence of autoantibodies and can encompass a broad range of conditions, including systemic lupus erythematosus (SLE), sjögren syndrome, systemic sclerosis (SSc), rheumatoid arthritis (RA), idiopathic inflammatory myopathies, mixed connective tissue disease, systemic vasculitis, and others^3,4^. Despite the recent advances in diagnosing, classifying, and treating SRDs, there are still significant challenges that need to be addressed. The standard treatment for management includes various forms of immunosuppression, however, not all patients respond effectively to these treatments, and sustained drug therapy is essential to maintain remission^5,6^.

Chimeric antigen receptor (CAR) T cell therapy is a recent and rapidly developing treatment modality, which involves the genetic engineering of a patient’s own T cells while using a retrovirus or lentivirus to introduce a CAR fusion protein^7,8^. These CAR T cells are then able to target and destroy cells that express a specific antigen, leading to strong T cell activation resulting in potent antitumor responses. This therapy has shown remarkable response in hematological malignancies such as lymphoma, leukemia, and multiple myeloma, as well as some solid tumors, with the possibility of long-term remission or even cure in some patients^8–11^.

Although initially developed for the treatment of cancer, studies have shown CAR T cells can be a promising new treatment option for autoimmune diseases as they have the ability to target and delete pathologically activated immune cells or re-establish tolerance in organs affected by dysregulated immunity^12–14^. This is of great therapeutic interest in autoimmune diseases as these conditions are characterized by abnormal immune responses that lead to chronic inflammation and tissue damage^14^. By using CAR T cells to target and eliminate specific cells that are contributing to the autoimmune response, it may be possible to reduce inflammation and improve outcomes for patients. However, this is still an emerging area of research and more studies are needed to evaluate the safety and efficacy of CAR T cells in autoimmune diseases. Thus, in this review, we aim to summarize and evaluate the current state of research on the use of CAR T therapy in the management of SRDs, focusing on the exploration of the potential benefits and limitations of CAR T therapy in the treatment of SRDs.

## Discussion

The first successful application of lymphocytes in tumor treatment was observed in mice in the 1980s, but it took until 2017 for a similar successful outcome in humans^15–17^. After the successful results seen with Tisagenlecleucel-T, the first CAR T cell therapy approved by the FDA for patients under 25 with B cell acute lymphoblastic leukemia (60% complete remission rate, 81% overall response rate), its use was expanded to treat other diseases, including SRDs^10,13,14,18,19^.

The current treatments for SRDs have certain limitations, with elevated susceptibility to infections, the requirement for periodic injections, and the development of anti-drug antibodies leading to treatment failure or adverse reactions^14^. CAR T cell therapy in autoimmune diseases involves targeting autoantigenic B cells, which cause autoimmune reactions, without affecting the entire immune system, similar to how it is used to treat cancer^20^. The CAR molecules are made up of an extracellular domain, composed of a single chain variable fragment of an antibody, which provides specificity for the specific cancer cell antigen, and an intracellular domain derived from T cell receptor (TCR) signaling proteins. The CARs are inserted into CD8+ T cells using viral vectors and redirected to identify and target the cells, activating the lytic mechanism of T cells and leading to its lysis^21,22^. CAR T cells targeting CD19 have been shown to induce comprehensive B cell aplasia with a single administration, preserve pre-existing humoral immunity, and specifically eliminate pathogenic B cells^23,24^. It is a promising approach for treating autoimmune diseases due to its ability to provide specific and long-lasting remission^25,26^.

There have been studies conducted to assess the use of CAR T cells in autoimmune diseases, such as in the case of pemphigus vulgaris where CAR T cells successfully eliminated B cells causing the disease^20,26,27^ . Moreover, studies have also described the use of two different CAR T cell approaches for the treatment of autoimmune diabetes in which a CAR molecule was designed based on an antibody that recognizes the major histocompatibility complex (MHC) class II: peptide complex, which was linked to Cluster of Differentiation (CD28, CD137, and CD247) activation and signaling domains. The CAR was used to target antigen presenting cells involved in stimulating autoimmune CD4+ T cells^28^. Another study explored the use of CAR T cells that targeted autoimmune CD8+ T cells using an MHC class I CAR linked to antigenic peptide/Beta-2-Microglobulin (β2m)/CD3ζ domains^29^. All of these studies demonstrated the potential of CAR T cell therapy as a new treatment option for autoimmune diabetes.

However, its application in SRDs is found to be limited because a single CAR T cell cannot effectively target the different types of autoreactive lymphocytes present in patients with SRDs^13^. Moreover, the challenge in using single chain variable fragment CAR T cell therapy for autoimmune diseases lies in identifying a specific cellular target for the pathogenic CD4+ T cells^18^. This involves identifying a target that specifically marks the harmful cells and is not present on healthy cells, to prevent harm to normal tissues. To overcome this limitation, studies have taken up approaches, which aim to create a universal CAR T cell therapy by detecting the presence of autoreactive lymphocytes and targeting them with a single therapy^13,18^. The idea is to identify the levels of autoantibodies present in a patient and target the major epitope peptides to create a therapy that can be used in a broader patient population^13^. However, this is still an active area of research and further studies are needed to fully understand the feasibility and effectiveness of this approach.

Adoptive transfer of CAR-Tregs is another mechanism for the use of CAR T technology for autoimmune diseases^30,31^. CAR-Tregs have the potential to effectively reduce inflammation, induce transplant tolerance, prevent graft versus host disease, and treat autoimmunity^32–34^. However, careful consideration must be given to the source of therapeutic cells, antigen-specificity, and homing characteristics to maximize the efficacy of Treg therapy. The isolation and expansion of Tregs from patients with autoimmune disease is feasible, but the achievement of clinically relevant Treg numbers may still be challenging in clinical use^31^.

CAR T cell design has the potential for flexibility and utility in treating various diseases, however, the optimal signaling domains are likely to be variable. Advances in regulating CAR T cells after adoptive transfer, both in terms of activation and deactivation, offer significant potential for creating highly effective immunotherapies^18^.

In the following text, we attempt to review and analyze existing studies to determine the potential, feasibility, effectiveness, and safety of CAR T therapy for the management of SRDs. Our goal is to provide a comprehensive overview of the current state of research and identify areas for future study in order to advance the use of CAR T therapy in the management of SRDs.

## The use of CAR T in systemic lupus erythematosus

SLE is a systemic autoimmune disease in which the immune tolerance against nuclear antigens including double-stranded (ds) DNA and nuclear proteins is broken, leading to the emergence of autoantibodies against dsDNA, and other nuclear antigens, which subsequently trigger immune complex-induced inflammation in an array of different organs, such as the kidneys, the heart, the lungs, and the skin^12^.

Despite the tremendous progress in treatment options, including monoclonal antibodies that deplete CD20 B cells, there are some patients who do not respond to the current state-of-the-art treatment for SLE, resulting in organ injury and even death. Treatment failures have been attributed to a lack of sustained/insufficient depletion of B cells, inaccessibility of affected tissues, and a lack of expression of CD20 on long-lived plasma cells and plasmablasts that produce antinuclear antibodies.^35^

CAR T cell therapy is of high interest, especially in cancer therapies, however, it has been gaining attraction in autoimmune conditions, including SLE. The prospect of highly effective depletion of target cells, CD 19 B cells in the context of SLE, through activated T cells sounds promising. It was first studied by Kansal *et al.* in murine lupus. They used purified CD8+ T cells due to the potential disease-enhancing effect of autoreactive CD4+ T cells. The recipient mice were conditioned with radiation to facilitate T cell engraftment. This resulted in increased life spans of two mouse strains, with most CD19-d NZB/W mice living up to 18 months after treatment, compared to the untreated group, which had all been euthanized at that point. Eight out of 12 CAR T cell treated MRL-Ipr mice lived for 1 year, reaching 14 months of age, surpassing the control group. This showed that CAR T cell therapy significantly impacted the progression of autoimmune disease in both strains of mice. They also measured proteinuria in lupus mice and found that 5 months after CAR T cell administration, none of the treated mice had high-grade proteinuria in comparison to the control group. They also reported a decrease in the ratio of CD4/CD8 seen in the control group along with a decrease in CD19 expression in the spleen, kidneys, and bone. They showed that the CD19-directed CAR T cell remained viable and functional several months after injection along with the effective elimination of autoantibody production. The authors suggested that CD19-targeted CAR T cells are more effective than antibody-mediated cytotoxicity because the former directly kills target cells, while the latter requires the accumulation of bound antibodies for target cell lysis or clearance by phagocytes. In autoimmune diseases, the high levels of endogenous antibodies and immune complexes can impair the depletion of B cells by macrophages. In contrast, CAR T cells can kill B cells effectively without the need for an accessory cell type^26^.

In a study by Mackensen *et al.*, five patients with the SLEDAI-2K (with Systemic Lupus Erythematosus Disease Activity Index-2000) score of 8–16 who had previously failed multiple therapies were enrolled, which showed a significant decrease in the SLEDAI-2K score, including active urine sediments and proteinuria, nephritis, and an increase in complement levels at the end of 3 months. Other severe manifestations (arthritis, fatigue, fibrosis of cardiac valves, and lung involvement (restriction and diffusion impairment) also improved. Anti-ds DNA levels dropped below the cutoff and INF-alpha was undetectable. All immunomodulatory agents were discontinued achieving drug-free remission in all five patients. At long-term follow-up, B cell reconstitution was observed in the five patients without a relapse of SLE. They also did not find a substantial decline in vaccine responses indicating that CAR T therapy primarily affected auto-antibody-producing cells rather than all immunoglobulin-producing cells. Cytokine release syndrome, considered a frequently observed toxicity with this therapy, was only seen in the mild form. Immune effector cell-associated neurotoxicity syndrome was not observed and no infection was noted during the treatment duration either^12^.

Salmon *et al.* discussed that in the study by Mackensen *et al.*, the five patients had low-tier or absent anti-RBP antibodies at baseline and postulated that in patients with pathogenic autoantibodies: for example, anti-RBP produced by long-lived plasma cells, will probably require a different kind of therapy than the one described by Mackensen *et al.* Salmon^35^ also suggested that although this study showed a B cell ‘reset’ with repopulating B cells expressing IgM/IgD over Immunoglobulin G/IgA in the short-term, during the long-term it is possible that B cells with a move down the differentiation pathway and resume making autoantibodies.

Kretschmann *et al.* ^36^ tested the functionality of anti-CD-19 CAR T cell therapy in patients with SLE after tapering glucocorticoids and stopping T cell suppressive drugs, which showed high expansion rates and viability sufficient and effective for clinical use.

## The use of CAR T cell therapy in rheumatoid arthritis

RA is an autoimmune disorder that causes chronic inflammation in the joints, resulting in joint damage^37^. The underlying causes and mechanisms of the disease are not entirely clear, however, research suggests that the process of citrullination may play a role^37,38^. The presence of anticitrullinated protein antibodies in the blood is very specific for RA and is associated with the disease’s severity and progression^38,39^. RA is a gradually progressive disease and without proper treatment, it can lead to irreversible joint damage, which impacts physical and emotional well-being, as well as increased mortality secondary to complications and comorbidities^40,41^.

Advancements in pharmaceuticals have led to new therapeutic approaches for treating RA, but a lack of understanding of molecular mechanisms is a challenge in finding a cure. Conventional synthetic DMARDs, biologic DMARDs, and targeted synthetic DMARDs are available to maintain joint function, but their use is associated with many significant side effects and require careful monitoring^42^. Targeted synthetic DMARDs, such as JAK inhibitors, have shown promising results as a newer class of drugs^43^. NSAIDs and glucocorticoids are also used as additional treatments to relieve inflammation and pain^44^. The aim of therapy in RA is to reach a state of remission and minimize any potential harmful side effects^45^.

Despite the presence of available treatments, a significant number of RA patients still do not respond to the current medications. Thus, the development of new therapies and adopting a more personalized approach to treatment becomes very important^46^. The successful implementation of CAR T cell therapy in 2017 has spurred on a plethora of studies to examine its efficacy in treating RA. However, the utilization of CAR T cell therapy for RA like other SRDs is challenged by its limited specificity to a single cell type, rendering it insufficient in effectively addressing the heterogeneous population of autoreactive lymphocytes present in RA patients^13^.

To overcome this limitation, Zhang *et al.* in 2020 published a proof of concept study which used an universal antifluorescein isothiocyanate (FITC) CAR T cells that were combined with FITC-labeled RA-immunodominant peptides. This study showed that multiple strains of hybridoma cells can be targeted and eliminated by the anti-FITC CAR T cells via lysis, depending on the availability of the relevant FITC-labeled antigenic peptides, offering a solution for the diverse nature of RA treatment with CAR T cells. This approach aimed to specifically eliminate different types of autoreactive B cell subsets, providing a more selective and persistent treatment option for patients with RA^13^.

Moreover, this study also tested for off-target effects of anti-FITC CAR T cells and found no significant toxicity to FcγR+Raw264.7 cells except when an excessive amount of a specific antibody was added. These results suggest that the off-target toxicity is unlikely and primarily only caused by low-avidity antibodies like ACPA, which account for a small fraction of total Immunoglobulin G. Moreover, no significant cytotoxic activity was observed for the control groups, showing the high selectivity of this approach and suggesting its potential to target pathogenic autoimmune cells without affecting protective immunity^13^.

The main limitation of the study is that it only demonstrated the elimination of autoreactive B cells in vitro and lacked evidence of the therapeutic effects of the approach in vivo. Another challenge is the stability of the peptide-based mediator^13^. This proof of concept study is still a significant step forward in the field of targeted treatment for systemic autoimmune diseases and opens up opportunities for additional research and development in this area.

Another study by Whittington *et al.* utilized an approach to treat autoimmune diseases which targets only the pathogenic CD4+ T cells responsible for the autoimmune disease pathology by incorporating the HLA-DRB1×01:01 (DR1) and a model autoantigen as part of the CAR molecular structure. The resulting DR1 CAR T cells lyse CD4+ T cells in an antigen-specific manner, only targeting cells expressing a TCR restricted to DR1 and specific for the antigenic peptide^18^. Studies in a humanized mouse model of RA using DR1- Type II Collagen (CII) CAR T cells showed that they effectively identified and lyse CII-specific CD4+ T cells and reduced the autoimmune T cell response and the severity of RA in vivo. The DR1 CAR T cells also reduced the severity of RA, as well as the B cell autoantibody response^18^. The specificity of the CAR T cell can be reprogrammed by changing the antigenic peptide sequence works as an advantage for its use in autoimmune diseases. These results suggest that MHC class II-based CAR T cells could have potential for treating autoimmune diseases in an antigen-specific manner.

The DR1 CAR T cells were found to maintain their cytolytic function over 90 days in culture, even with the expression of markers associated with CD8 T cell exhaustion. However, although they showed function in vivo, they were unable to persist as a memory phenotype due to the low frequency of target cells and the lower affinity of the DR1 CAR for the target TCR. The frequency of antigen-stimulated CD4+ T cells was found to be low, and the affinity of MHC class II binding by TCR was lower than that of antibodies. Continuous stimulation of the DR1 CAR T cell through the DR1 CAR was insufficient to maintain the cells in culture over time. As such, new signaling domains have been developed for CARs to improve clinical efficacy and may be beneficial for DR1 CAR function^18^.

Immunodepletion has been found to enhance the efficacy of CAR T cell therapy for autoimmune diseases^47^. In patients with autoimmune disorders, immunodepletion could be used prior to CAR T cell treatment for better results, as it could provide significant benefit in treating the disease. However, immunodepletion was not used in the studies mentioned due to its potential interference with the development of autoimmune arthritis and the interpretation of CAR T cell treatment data^18^. This is an aspect that could be examined in prior research and future studies on the application of CAR T cell therapy for autoimmune conditions.

Targeting citrullinated vimentin (CV), which is a specific antigen found exclusively in the inflamed synovial tissue of RA patients can be a potential target for CAR T cell therapy^48^. Recent research has explored the potential use of engineered CAR-Tregs (T cells modified with a CAR) to target CV in RA. Preliminary, unpublished data suggest that CAR-Tregs directed against CV-CAR-Tregs may react with CV expressed in RA synovial fluid^14^. However, further studies are needed to investigate the effect of CV-CAR-Tregs in preclinical RA.

## The use of CAR T cell therapy in systemic sclerosis

SSc is a chronic autoimmune disease characterized by widespread fibrosis and vasculopathy of multiple organs, including the skin and lungs^49^. The symptoms, progression, and treatment response in SSc can vary greatly from person to person, and the underlying cause of the disease remains unknown. Studies have found that treatment with cyclophosphamide, mycophenolate, and hematopoietic stem cell transplant can effectively improve cutaneous sclerosis^50^. In recent years, significant progress has been made in controlling the vascular component of SSc, and nintedanib and tocilizumab have been approved for the treatment of interstitial lung disease associated with SSc^50,51^. However, more effective therapies are still needed, especially those with broader disease-modifying properties.

CAR T cell therapy is a promising approach for the treatment of SSc, particularly in light of the dysregulation of B cell homeostasis in this disease and the positive results observed with rituximab^14,52^. B cells have been shown to play a role in the pathogenesis of SSc by producing autoantibodies and contributing to the chronic inflammation that leads to fibrosis^53^. As a result, targeting B cells responsible for producing pathogenic autoantibodies involved in the pathogenesis of SSc, such as antiendothelial or antifibroblast antibodies, could lead to the design of CAR T cells that specifically delete these cells while preserving protective B cells^14,53^.

In addition to the dysregulation of B cells, changes in the Treg population have also been observed in SSc patients^54^. There is evidence to suggest that the balance between Treg and Th17 cells is disrupted, leading to an overactive Th17 response and increased inflammation^54,55^. This immune imbalance is thought to contribute to the fibrosis and tissue damage seen in SSc patients^54–56^. As a result, therapies that target Treg or Th17 cells are being explored as potential treatments for SSc, with the goal of restoring the balance between these cell types and reducing inflammation^57^. Designing CAR-Tregs targeted to specific skin and lung antigens could allow for the induction of tolerance in the early and late phases of SSc, potentially alleviating inflammation and promoting physiologic tissue repair^14^. However, the effect of tolerance induction at different fibrotic stages requires further investigation.

Finding a specific myofibroblast protein that could be targeted by CAR T cells could provide a new and exciting approach for the treatment of SSc. Targeting fibroblast activation protein by CAR T cells has also shown promising results, inducing a significant reduction of cardiac fibrosis and restoration of function after injury in mice. Results showed that fibroblast activation protein was expressed in failing human hearts and was a potential target for T cell therapy. No significant toxicities were observed in non-cardiac organs or tissues and no significant increase in cytokine levels was seen. However, the study also found some gene expression changes that were only partially consistent with cardiotoxicity^19^. Further research is needed to fully understand the potential benefits and risks of this therapy.

## The use of CAR T in juvenile idiopathic arthritis and juvenile dermatomyositis

Juvenile idiopathic arthritis (JIA) and juvenile dermatomyositis (JDM) are autoimmune disorders that affect children. JIA encompasses several distinct conditions that can cause joint inflammation in children and teenagers, starting before the age of 16 that causes joint pain, swelling, and stiffness, leading to joint damage, and loss of mobility^58^. JDM, on the other hand, is a rare autoimmune disease that affects the muscles and skin, causing weakness and a distinctive rash^59^. Both JIA and JDM can have a significant impact on a child’s quality of life and can lead to long-term disability if left untreated. The current treatment options for these autoimmune disorders include immunosuppressants, but these treatments can also lead to serious side effects^60,61^.

This is where CAR-Tregs offer a promising alternative. CAR allows the Tregs to specifically target cells that express the antigen of interest, leading to a more selective immune response and a reduction in inflammation. The goal of generating CAR-Tregs for treating JIA and JDM is to modulate the immune response in a way that reduces inflammation and tissue damage without causing widespread immunosuppression^30^. As the specific antigen causing the autoimmune response is not always known in pediatric JIA and JDM patients, the generation of antigen-specific Treg cells and achieving clinically relevant Treg numbers may be challenging^31^.

However, CAR-Tregs that target antigens at the site of inflammation have shown potential, as have approaches that condition Tregs in vitro to specific sites as have approaches that inject Tregs locally at the site of inflammation. The use of CAR-Tregs that can react with antigens found at the site of inflammation could allow for local activation of Tregs^31^. Another approach could be to condition Tregs in vitro to target specific sites, or to inject Tregs locally as has been shown in murine studies for allograft skin inflammation^62,63^. In Table 1 we have summarized the potential use of CAR T therapy with its advantages over traditional therapy in SRDs.

**Table 1:** Summary of potential use of CAR T therapy with advantages over traditional therapy in systemic rheumatic diseases

Systemic rheumatic diseases	CAR T cells used	Potential application	Advantages over traditional therapy
Systemic Lupus Erythematosus (SLE)	Anti-CD-19 CAR T cell	Refractory SLE	CAR T cells remained viable and functional several months after injection. CAR T cells were found to kill B cells effectively without the need for an accessory cell type unlike macrophages. CAR T cells were seen to induce remission, prolong lifespan, decrease the progression of nephritis in murine models with minimal adverse effects.
Rheumatoid Arthritis (RA)	Antifluorescein isothiocyanate (FITC) CAR T cells HLA-DRB1×01:01 (DR1) CAR T cells	Treatment Failure	Multiple strains could be eliminated by the anti-FITC CAR T cells via lysis offering an universal treatment solution for the diverse nature of RA with limited toxicity. DR1 CAR T cells only target CII-specific CD4+ T cells and reduce the autoimmune T cell response and the severity of RA.
Systemic Sclerosis (SSc)	CAR-Tregs targeted to specific skin and lung antigens: example: Fibroblast Activation Protein (FAP)	To control the development fibrosis in SSc	Anti FAP CAR T cells have shown to significantly reduce cardiac fibrosis and restoration of function after in mice with insignificant toxicity.
Others Juvenile Idiopathic Arthritis (JIA) Juvenile Dermatomyositis (JDM)	CAR-Tregs	Local application at the site of inflammation	Potential of CAR-Tregs in modulating the immune response in a way that reduces inflammation and tissue damage without causing widespread immunosuppression in children and teenagers.

## Conclusion

Our review has demonstrated the potential of CAR T in the treatment of SLE, RA, SSc, JIA, and JDM. However, further studies with a bigger sample size are required to fully understand its clinical efficacy, safety, long-term effects and whether disease remission can be sustained beyond the remission periods. There is also a lot to learn about the optimal design, dosing, and delivery of CAR T cells along with the technical demand and expenses associated with this therapy. Thus, research is also needed to determine its effectiveness in relation to cytotoxic conditioning regimen and anti-CD-19 monoclonal antibody therapy. As SRDs have multiple associated antigens, it is challenging to identify the best cellular antigens to pursue. Thus, a universal therapy that can be used across a broader patient population is also needed. While early studies have shown promise, additional research will be necessary to determine whether this approach can deliver meaningful benefits for patients with SRDs.

## Limitations

The inclusion criteria and the authors’ perspectives may influence the selection and interpretation of studies, which could limit the generalization of the findings. As a result, this review may not provide a comprehensive or fully representative overview of CAR T cell therapy for the new topic at hand.

## Ethical approval

NA.

## Consent

NA.

## Sources of funding

None.

## Author contribution

S.B.: concept, manuscript writing, review; S.B.: concept, manuscript writing, review; S.B.: manuscript writing, editing, review.

## Research registration unique identifying number (UIN)

Not applicable.

## Conflicts of interest disclosure

All the authors declare no conflicts of interest.

## Guarantor

Dr Sadikshya Bhandari, Dhulikhel Hospital, Kathmandu University School of Medical Sciences Dhulikhel, Kavre, Nepal. E-mail: Bhandarisadikshya96@gmail.com.

## Data availability statement

Not applicable.

## Provenance and peer review

Not commissioned, externally peer reviewed.
